# Brief diesel exhaust exposure acutely impairs functional brain connectivity in humans: a randomized controlled crossover study

**DOI:** 10.1186/s12940-023-00961-4

**Published:** 2023-01-14

**Authors:** Jodie R. Gawryluk, Daniela J. Polombo, Jason Curran, Ashleigh Parker, Chris Carlsten

**Affiliations:** 1 Department of Psychology, Division of Medical Sciences, University of Victoria, 3800 Finnerty Road, BC V8P 5C2 Victoria, Canada; 2 Department of Psychology, University of British Columbia, 2329 West Mall, BC V6T 1Z4 Vancouver, Canada; 3 Air Pollution Exposure Laboratory, Respiratory Medicine, University of British Columbia, The Lung Centre, 2775 Laurel Street, 7th Floor, BC V5Z 1M9 Vancouver, Canada; 4 Department of Psychology, University of Victoria, 3800 Finnerty Road, BC V8P 5C2 Victoria, Canada

**Keywords:** Air pollution, Functional magnetic resonance imaging (MRI), Controlled human exposure, Environmental health, Neuroimaging

## Abstract

**Background:**

While it is known that exposure to traffic-related air pollution causes an enormous global toll on human health, neurobiological underpinnings therein remain elusive. The study addresses this gap in knowledge.

**Methods:**

We performed the first controlled human exposure study using functional MRI with an efficient order-randomized double-blind crossover study of diesel exhaust (DE) and control (filtered air; FA) in 25 healthy adults (14 males, 11 females; 19–49 years old; no withdrawals). Analyses were carried out using a mixed effects model in FLAME. Z (Gaussianised T/F) statistic images were thresholded non-parametrically using clusters determined by Z > 2.3 and a (corrected) cluster significance threshold of *p* = 0.05.

**Results:**

All 25 adults went through the exposures and functional MRI imaging were collected. Exposure to DE yielded a decrease in functional connectivity compared to exposure to FA, shown through the comparison of DE and FA in post-exposure measurement of functional connectivity.

**Conclusion:**

We observed short-term pollution-attributable decrements in default mode network functional connectivity. Decrements in brain connectivity causes many detrimental effects to the human body so this finding should guide policy change in air pollution exposure regulation.

**Trial registration:**

University of British Columbia Clinical Research Ethics Board (# H12-03025), Vancouver Coastal Health Ethics Board (# V12-03025), and Health Canada’s Research Ethics Board (# 2012-0040).

## Background

Exposure to traffic-related air pollution (TRAP) has long been associated with a range of adverse health effects, principally cardiovascular and respiratory [1]. This poses an enormous global burden, in terms of morbidity and lost productivity, as well as deaths estimated at approximately five million per year worldwide [2]. This profound toll is increasingly appreciated as including impacts on the central nervous system, but the data therein remains immature. Further, neurobiological underpinnings of these observations remain elusive, although some preliminary data suggest direct transmission of particles via the olfactory bulb and/or secondary transmission of inflammation likely generated more proximally [3–5]. Given the profound implications for public health across essentially all communities [6], data that adds overall biologic plausibility and also specific evidence of affected body systems are critically needed in order to support observational data [7–10]. Therefore, we performed the first controlled human exposure study to TRAP, using an established and safe paradigm of diluted diesel exhaust, that examines changes in functional MRI in an efficient crossover study (namely, diesel exhaust [DE] or filtered air [FA] exposure following light exercise), allowing observation of short-term effects on brain connectivity in this context.

## Methods

### Participants

A total of 100 MRI acquisitions were obtained in the current study. Twenty-five adult participants were tested immediately pre- and post-exposure to diesel exhaust (DE) and immediately pre- and post-exposure to filtered air (FA) for comparison. All participants were recruited through posters in the community, online notices, and e-mail notifications to the Vancouver Coastal Health Staff List-Serve.

Inclusion criteria for participants were as follows: between 19 and 49 years of age, able to converse in English, healthy, non-smoking, not pregnant or breast-feeding, and without any contraindications for MRI. So long as inclusion criteria were met, the only exclusion criteria was claustrophobia.

### Procedure

The study employed a controlled, double-blinded crossover design at the Air Pollution Exposure Lab. Each participant was tested in both the control condition (exposure to FA) and the experimental condition (exposure to DE) with four data acquisitions: (1) pre-FA; (2) post-FA; (3) pre-DE and (4) post-DE. The order of exposure to FA and DE was randomized and counterbalanced across participants, with a two-week delay between conditions. Both participants and individuals involved in collecting the MRI data were blinded to the condition, a technique that has been shown not only nominal but also effective [11].

FA or DE (nominal concentration: 300 µg of particulate matter of 2.5 microns or less [PM_2.5_]/m^3^) exposure occurred for 120 min [12]. During exposure, participants cycled on a stationary bicycle at light effort (that which yields ventilation at 15 L/min/m^2^) for 15 min, during the first quarter of each hour, to maintain a representative level of activity.

### Image acquisition

The following MRI protocol was employed pre-FA, post-FA, pre-DE and post-DE for each participant. All images were acquired at BC Children’s Hospital on a 3 Tesla GE Discovery MR750 MRI scanner. A whole-brain anatomical MRI scan was acquired with a T1-weighted FSPGR 3D sequence, with the following parameters: a repetition time (TR) of 8.148 ms, an echo time of 3.172 ms, voxel size of 1 × 1 × 1 mm, and a flip angle of 8°. A functional MRI (fMRI) scan was obtained during resting state (with eyes open or closed). The resting state fMRI scan was 6 min in duration and obtained with a T2*-weighted echo-planar imaging sequence with the following parameters: a repetition time of 2000 ms, an echo time of 19 ms, 180 volumes, 39 slices, and a voxel size of 3 × 3 × 3 mm. Additional task-based scans were obtained following resting state scans, but were ancillary to the hypotheses being tested here.

### Functional MRI data analyses

All analysis steps were performed using tools within the Functional MRI of the Brain Software Library (FSL; Analysis Group, FMRIB, Oxford, UK, http://fsl.fmrib.ox.ac.uk) [13]. Non-brain tissue in the raw T1 images was removed using the automated Brain Extraction Tool, followed by manual verification and optimization for each subject.

A seed-based approach was used to examine functional connectivity in the default mode network (DMN) [14]. The FEAT function was used to pre-process the data including skull removal (using the Brain Extraction Tool), motion correction (using MCFLIRT) [15], and highpass temporal filtering (using Gaussian-weighted least-squares straight line fitting with σ = 50.0 s). No smoothing was applied. Registration of the functional data to the high-resolution structural image was carried out using the boundary-based registration algorithm. Registration of the high-resolution structural images to standard space was carried out using FLIRT [15, 16] and then further refined using FLIRT or FNIRT nonlinear registration (optimized for each individual) [17, 18]. Next, the posterior cingulate cortex region of interest (ROI or seed) was registered to individual space. This ROI/seed was created based on ROIs from previous studies and included a 10 voxel spherical ROI centred on the following MNI coordinates: -2, -51, 27 [19, 20]. The FEAT function was used to examine the default mode network posterior cingulate cortex ROI/seed and to regress out the lateral ventricle signal to correct for confounding noise. Specifically, the mean blood oxygen level-dependent signal time series was extracted from the posterior cingulate seed region and used as the model response function in a general linear model analysis. This allowed for examination of functional connectivity in the DMN through the detection of voxels with timeseries that correlate with that measured in the posterior cingulate seed. The time-series statistical analysis was carried out using FILM (FMRIB’s Improved Linear Model) with local autocorrelation correction and correction for motion parameters [21].

Higher-level analyses were carried out using FMRIB’s Local Analysis of Mixed Effects (FLAME), an approach for multisubject and multisession fMRI data analyses [22, 23]. Specifically, this approach allowed for higher-level within group comparisons of resting state functional connectivity in the DMN pre- versus post-DE exposure, pre- versus post-FA exposure, pre-FA versus pre-DE exposure and post-FA versus post-DE exposure (all contrasts were examined bidirectionally). Z (Gaussianised T/F) statistic images were thresholded non-parametrically using clusters determined by Z > 2.3 and a (corrected) cluster significance threshold of *p* = 0.05 [22, 24] The pre-exposure MRI effectively serves as a baseline for a given individual and, given the crossover design of this study, each individual served as his/her own control, virtually eliminating the concern for confounding by personal characteristics [12].

## Results

In the present study, we focused on putative effects of TRAP on the default mode network (DMN), a set of inter-connected cortical brain regions in which activity is maximal at rest or during internal thought engagement. We focused on the DMN, given the preferential vulnerability of this network to aging [25, 26], toxicity [27], and disease states [28, 29].

The 25 participants were 11 female and 14 male, with mean age of 27.4 (s.d. 5.5) years. Exposure conditions were achieved as follows, in terms of PM_2.5_ as µg/m^3^, for filtered air (FA): 2.4 and for DE: 289.6; total volatile organic carbons (ppb) for FA: 124.5 and for DE: 1425.0; carbon dioxide (ppm) for FA: 794.1 and for DE: 2098.0; nitrogen dioxide (ppb) for FA: 51.9 and for DE: 283.1.

**Fig. 1:**
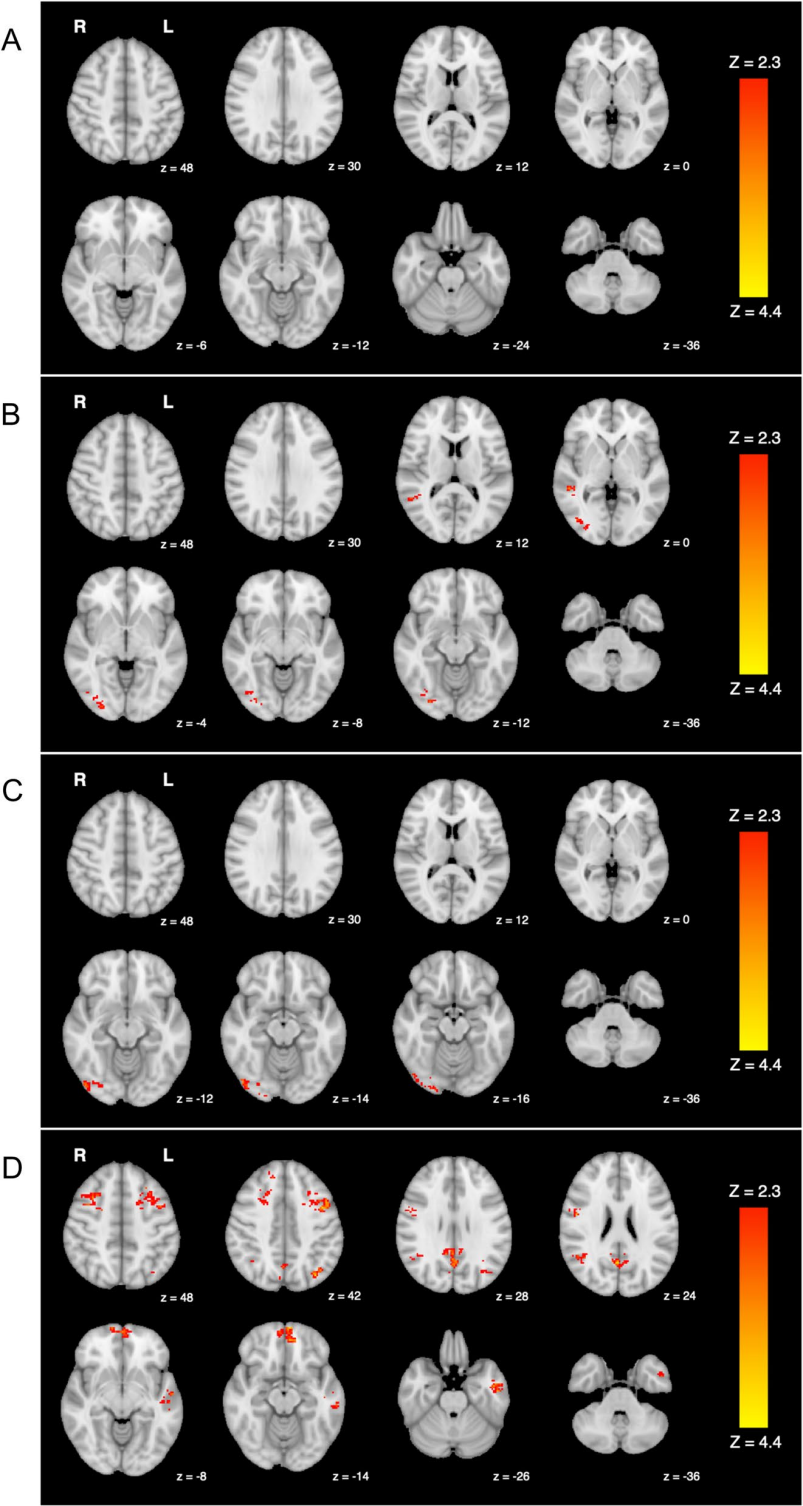
Results of group level comparisons (*p* < 0.05, corrected) with significant regions in red. **A** represents no significant findings pre- versus post- diesel exhaust. **B** depicts regions with increased functional connectivity post-filtered air > pre-filtered air. **C** shows regions with increased functional connectivity pre-diesel exhaust > pre-filtered air. **D** depicts areas with greater functional connectivity post-filtered air > post-diesel exhaust

In the DE group, there were no significant differences in DMN functional connectivity for post- compared to pre-DE exposure (Fig. 1A). By contrast, in the FA group, significantly greater DMN functional connectivity was observed post-exposure relative to pre-exposure, localized in the right middle temporal gyrus and occipital fusiform gyrus (Fig. 1B; Table 1).

**Table 1:** Functional connectivity post- and pre-filtered air exposure

Brain Region		MNI Coordinates			
	Laterality	X	Y	Z	Z score
Middle Temporal Gyrus	R	55	-50	6	2.61
Occipital Fusiform Gyrus	R	29	-80	-13	3.06

Brain regions showing increased functional connectivity in the post-filtered air condition relative to the pre-filtered air condition (min Z > 2.3; cluster significance: *p* < 0.05, corrected for multiple comparisons). Coordinates in the MNI-152 standard space image are provided

There were small albeit significant differences observed when comparing groups pre-exposure. Specifically, participants demonstrated greater functional connectivity, pre-DE compared to pre-FA, in the right occipital fusiform gyrus as well as the occipital pole (Fig. 1C; Table 2). However, a more robust pattern of significant differences emerged when groups were compared post-exposure. Participants demonstrated greater functional connectivity in widespread regions of the default mode network following exposure to FA compared to following exposure to DE (Fig. 1D; Table 3). Briefly stated another way, exposure to DE yielded a decrease in functional connectivity compared to exposure to FA.

**Table 2:** Functional connectivity pre-diesel exhaust exposure and pre-filtered air exposure

Brain Region		MNI Coordinates			
	Laterality	X	Y	Z	Z score
Occipital Fusiform Gyrus	R	28	-80	-13	2.90
Occipital Pole	R	22	-94	-13	2.38

Brain regions showing increased functional connectivity in the pre-diesel exhaust condition relative to the pre-filtered air condition (min Z > 2.3; cluster significance: *p* < 0.05, corrected for multiple comparisons). Coordinates in the MNI-152 standard space image are provided

**Table 3:** Functional connectivity post-diesel exhaust exposure and post-filtered air exposure

Brain Region		MNI Coordinates			
	Laterality	X	Y	Z	Z score
Angular Gyrus	R	51	-54	26	2.83
Frontal Pole	R	12	66	-8	3.40
Frontal Pole	L	-7	63	-14	4.20
Middle Frontal Gyrus	R	36	19	50	2.68
Middle Frontal Gyrus	L	-48	12	42	2.75
Middle Temporal Gyrus	R	-62	-23	-11	3.19
Middle Temporal Gyrus	L	-60	-27	-13	2.95
Precuneus Cortex	R	4	-60	25	3.83
Precuneus Cortex	L	-5	-58	36	3.54
Temporal Pole	L	-46	4	-38	2.68

Brain regions showing increased functional connectivity in the post-filtered air condition relative to the post-diesel exhaust condition (min Z > 2.3; cluster significance: *p* < 0.05, corrected for multiple comparisons). Coordinates in the MNI-152 standard space image are provided

## Discussion

Our study provides the first evidence in humans, from a controlled experiment, of altered brain network connectivity acutely induced by air pollution. The use of this model is important because it is not subject to potential confounding by variables correlated to exposure, a vexing concern common to observational studies. The precise functional impact of the changes seen in fMRI are unknown but are likely modest given the small magnitude of change, as expected with such limited exposure. That said, real-world exposures are often more persistent, particularly in regions of the world for which levels such as those we use are not uncommon. It is hypothesized that chronic exposure is effectively a series of short-term exposures (only one of which our participants were exposed to) that ultimately leads to accumulated deficits through a stress on allostatic load [30, 31], but whether or not this applies to pollution in the neurocognitive realm, while hypothesized [32], requires further study. That being said, our results are consistent with a study of chronic air pollution exposure in Germans [33].

In considering why DE attenuated functional connectivity in the DMN relative to FA, it is worth noting previous studies have demonstrated increased functional connectivity following exercise and the results for the FA condition are consistent with these findings [34, 35]. However, these results were only found when participants were exposed to the FA condition (whereas no significant change in functional connectivity was detected pre-post DE exposure). Therefore, our current results suggest that the brain-related benefits of light exercising (e.g., increased functional connectivity) are not obtained under the DE condition. Although previous observational investigations suggest exposure to air pollutants is associated with decreased functional connectivity [36, 37] the current results are an extension of these findings, given that the DE condition elicited a relative decrease in functional connectivity compared to the FA condition. Our demonstrating this using such directly controlled methodology adds considerably to the plausibility if these previous findings. More precise mechanisms have been elusive to date, though a link to neuroinflammation (difficult to measure directly in the intact human), potentially secondary to particle migration via the olfactory bulb as seen in animal models [38], seems likely.

There are several ways in which decrements in brain connectivity, such as those we demonstrated, might manifest in daily life. Changes in brain connectivity have been associated with decreased working memory [39] and behavioural performance [40], and deterioration in productivity at work (which is also associated with air pollution) [41]. It is also possible that these decrements worsen further in the context of multifaceted exposures not studied here [42].

## Conclusion

The current study represents the first controlled human exposure to diesel exhaust investigation using functional MRI. The results of an order-randomized double-blind crossover study of diesel exhaust and control air in healthy adults revealed immediate pollution-attributable declines in default mode network functional connectivity. Change in policy surrounding air pollution exposure has long been driven by a combination of observational and experimental evidence, which together are most compelling especially in the face of interests aggressively opposed to regulation that foster improved air quality. In spite of volumes of existing evidence regarding adverse effects of air pollution, history demonstrates that implicating additional organ systems can augment the already strong evidence and effectively apply further pressure for emissions control in areas lagging in that regard. This data may be informative therein, while deepening the evidence base for direct evidence of neurocognitive effects due to acute exposure to TRAP. As the changes in cognition we have demonstrated may put individuals at risk for impaired vocational performance, this is an important consideration for public health.

## Data Availability

The data generated during this study are available from the corresponding author (CC) on reasonable request.

## Abbreviations

DE: Diesel exhaust
DMN: Default mode network
FA: Filtered air
MRI: Magnetic resonance imaging
ROI: Region of interest
TRAP: Traffic-related air pollution
