# Correction: Brief diesel exhaust exposure acutely impairs functional brain connectivity in humans: a randomized controlled crossover study

**DOI:** 10.1186/s12940-023-00967-y

**Published:** 2023-01-23

**Authors:** Jodie R. Gawryluk, Daniela J. Palombo, Jason Curran, Ashleigh Parker, Chris Carlsten

**Affiliations:** 1 Department of Psychology, Division of Medical Sciences, University of Victoria, 3800 Finnerty Road, Victoria, BC V8P 5C2 Canada; 2 Department of Psychology, University of British Columbia, 2329 West Mall, Vancouver, BC V6T 1Z4 Canada; 3 Air Pollution Exposure Laboratory, Respiratory Medicine, University of British Columbia, The Lung Centre, 2775 Laurel Street, 7th Floor, Vancouver, BC V5Z 1M9 Canada; 4 Department of Psychology, University of Victoria, 3800 Finnerty Road, Victoria, BC V8P 5C2 Canada

**Correction: Environ Health 22, 7 (2023)**

**https://doi.org/10.1186/s12940-023-00961-4**

Following publication of the original article [1], the authors identified an error in the author name of Daniela J. Palombo.

The incorrect author name is: Daniela J. Polombo

The correct author name is: Daniela J. Palombo

The author group has been updated above and the original article [1] has been corrected.
